# Host Preference of Beneficial Commensals in a Microbially-Diverse Environment

**DOI:** 10.3389/fcimb.2022.795343

**Published:** 2022-06-15

**Authors:** Olga M. Pérez-Carrascal, Rebecca Choi, Méril Massot, Barbara Pees, Vivek Narayan, Michael Shapira

**Affiliations:** Department of Integrative Biology, University of California, Berkeley, Berkeley, CA, United States

**Keywords:** *C. elegans*, microbiota, *Enterobacteriaceae*, *Pantoea*, commensal, preference

## Abstract

Gut bacteria are often described by the neutral term commensals. However, the more we learn about their interactions with hosts, the more apparent it becomes that gut commensals often contribute positively to host physiology and fitness. Whether hosts can prefer beneficial bacteria, and how they do so, is not clear. This is of particular interest in the case of the bacterivore *C. elegans*, which depends on bacteria as food source, but also as gut colonizers that contribute to its physiology, from development to immunity. It is further unclear to what extent worms living in their microbially-diverse habitats can sense and distinguish between beneficial bacteria, food, and pathogens. Focusing on *Enterobacteriaceae* and members of closely related families, we isolated gut bacteria from worms raised in compost microcosms, as well as bacteria from the respective environments and evaluated their contributions to host development. Most isolates, from worms or from the surrounding environment, promoted faster development compared to the non-colonizing *E. coli* food strain. *Pantoea* strains further showed differential contributions of gut isolates versus an environmental isolate. Characterizing bacterial ability to hinder pathogenic colonization with *Pseudomonas aeruginosa*, supported the trend of *Pantoea* gut commensals being beneficial, in contrast to the environmental strain. Interestingly, worms were attracted to the beneficial *Pantoea* strains, preferring them over non-beneficial bacteria, including the environmental *Pantoea* strain. While our understanding of the mechanisms underlying these host-microbe interactions are still rudimentary, the results suggest that hosts can sense and prefer beneficial commensals.

## Introduction

Bacterial community composition in plant and animal hosts are typically distinct from those in the surrounding environments (Backhed et al., 2005; Hacquard et al., 2015; Wippel et al., 2021). What determines this distinction, and which factors dominate the process of microbiota assembly is an active area of research and a topic of debate (Ley et al., 2008; Gilbert et al., 2018; Sieber et al., 2019). Environmental availability and external environmental factors, such as temperature, toxins and host diet are important (Spor et al., 2011; Smith et al., 2015; Callens et al., 2018; Simon et al., 2019). Equally important is host filtering, which is dependent on host genetics and is dominated by host immunity, internal nutrient availability and behavior (Hacquard et al., 2015; Berg et al., 2016a; 2019; Davenport et al., 2017; Goodrich et al., 2017), but is also affected by bacterial interactions, which can impact microbiota diversity and are known to affect host physiology and fitness (Lawley and Walker, 2013; Simon et al., 2019; Johnke et al., 2020).

The nematode *C. elegans* is well-suited for studying host-microbe interactions. Clonal populations and tight genetic control reduce inter-individual variation when examining gut microbiota composition, and a transparent body permits *in vivo* monitoring of bacterial colonization (Shapira, 2017). Over the past several years, work in this system has yielded important insights into the processes that shape the gut microbiota, in particular with respect to the role of host genes (Berg et al., 2019; Ortiz et al., 2021; Zhang et al., 2021). *C. elegans* feeds on bacteria from its environment, but specific bacteria resist digestion and persist in the worm gut giving rise to a characteristic gut microbiota shaped by environmental availability as well as by host genetics (Berg et al., 2016a; 2016b; Zhang et al., 2017; 2021). Work in the past several years has characterized the composition and diversity of the *C. elegans* gut microbiota, and common taxa were identified across worm populations isolated from different geographical locations (Berg et al., 2016a; Dirksen et al., 2016; Samuel et al., 2016). Bacteria of the *Enterobacteriaceae* family are among the most prevalent, and several species of this family were shown to have positive effects on host development (Berg et al., 2019; Dirksen et al., 2020) [although this may come with a trade-off for adult stress resistance (Slowinski et al., 2020)]. Additional families including *Pseudomonadaceae*, *Xanthomondaceae*, *Comamonadaceae* and *Brucellaceae*, contain members that have similar contributions to worm development (Watson et al., 2014; Samuel et al., 2016; Dirksen et al., 2020; Zhang et al., 2021).

Gut bacteria are often described as commensals, but the more they are studied, the clearer it becomes that many contribute positively to host functions, such as development, as described above, metabolism (Zimmermann et al., 2020), and often, to colonization resistance - preventing invasion by pathogens, but also by non-pathogenic colonizers that may perturb homeostasis. Such colonization resistance depends on competition for nutrients, inter-bacterial warfare mediated by secreted toxins, or activation of host immunity (Lawley and Walker, 2013; Sorbara and Pamer, 2019). In *C. elegans*, colonization resistance has been so far shown to depend on secretion of anti-microbial peptides (Dirksen et al., 2016; Kissoyan et al., 2019) and on immune priming (Montalvo-Katz et al., 2013; Berg et al., 2016b; Willis et al., 2021). While research focuses on beneficial commensals, not all environmentally available bacteria are necessarily beneficial. Whether hosts can distinguish between species or strains that are beneficial and those that are less so is unclear. Previous work with *Enterobacter* commensals isolated from *C. elegans* or from *C. briggsae* demonstrated that commensals of one host species could protect it from a subsequent exposure to a pathogen, but failed to protect the other host species, suggesting a functional adaptation between hosts and their cognate commensals, the basis for which is still unknown (Berg et al., 2016b). If host preference for certain bacteria existed, it could be mediated by differential bacterial abilities to colonize and persist in the worm gut, or alternatively, by host behavior, as previously described for *C. elegans* preference of food sources that better supported its growth (Shtonda and Avery, 2006; Kim and Flavell, 2020).

In this study, we set out to expand the repertoire of beneficial gut bacteria, focusing on members of the *Enterobacteriaceae* family and of the *Enterobacterales* order, to examine whether some taxa are more likely than others to colonize the worm gut, and to explore the worm’s ability to prefer beneficial ones. This analysis identified the genus *Pantoea* [part of the *Erwiniaceae* family, a recent splinter off the *Enterobacteriaceae* family (Adeolu et al., 2016)], of which gut isolates showed significant contributions to host fitness. Further analysis demonstrated that beneficial *Pantoea* strains were preferred as gut commensals due to a greater ability to colonize than a non-beneficial congeneric, but also through host attraction toward these bacteria.

## Materials and Methods

### Standard Worm and Bacterial Strains

All experiments were performed using worms of the Bristol N2 strain. Those were raised either on nematode growth medium (NGM) or on peptone-free medium (PFM). *E. coli* strain OP50 was used as the standard food to raise worms and as a reference point in comparisons with other bacteria. A GFP-expressing version of *P. aeruginosa* strain PA14 was used as a model pathogen. The N2, OP50 and PA14-GFP strains were originally obtained from the *Caenorhabditis* Genome Center (CGC).

### Worm Growth in Compost Microcosms and Bacterial Isolation

Microcosms were prepared with 5 gr oak knoll soil composted for two weeks either with chopped bananas or apples, cured of endogenous non-*C. elegans* nematodes and reconstituted with the original bacterial community, as previously described (Berg et al., 2016a). Initially germ-free L1 larvae were added to microcosms, raised at 20°C for four days, following which adults were harvested, washed intensively and surface sterilized, ascertaining removal of external bacteria by plating final wash solutions on lysogeny broth (LB) plates/no antibiotics (Berg et al., 2016a). Bacteria were released from worms by grinding the latter with a motorized pestle in a volume of 100 µl M9, pelleting debris, and plating bacteria from supernatants on *Enterobacteriaceae/Enterobacterales*-selective violet red bile glucose (VRBG) agar (Difco) or on non-selective rich media, tryptic soy (TS) or LB agar. Plates were incubated at 25°C for 48 hours, from which colonies were regrown, and frozen at -80°C till subsequent analyses. Bacteria were similarly cultured from the respective microcosm environments, following resuspension in M9 solution, vortexing to disrupt larger soil aggregates and plating supernatant after spinning down (one minute at 1800 rpm) soil particles.

Twenty bacterial clones were randomly selected from each microcosm environments. Approximately 160 strains were isolated in total, of which 69 (41 from soil, 28 from worms) were taxonomically classified at the genus level following Sanger sequencing of full-length 16S rRNA gene amplified using primers 27F (5′-AGA GTT TGA TCC TGG CTC AG-3′) and 1492R (5′-GGT TAC CTT GTT ACG ACT T-3′).

### Development Rate Measurements

The impact of individual bacterial strains on *C. elegans* growth rate was assessed by comparing the percentage of animals reaching larval L4 stage to that in animals raised on *E. coli* OP50. Each strain was tested in at least two independent experiments. Worm populations were raised from eggs obtained by bleaching gravid worms, or from starvation-synchronized L1 larvae (50-100 worms per plate), on lawns of bacterial isolates (cultured overnight in LB at 28°C, concentrated 10x and plated on NGM plates) for 40 hours at 25°C, at which point the percentage of L4 larvae was evaluated. Each plate was divided to quarters. Starting to the upper left of the quadrant, the stage of the first 25 worms observed was assessed.

### Preventing Infective Colonization and Fluorescent Imaging

L1 worms were grown at 25°C to early adulthood (36 hours) on PFM plates with lawns of different *Pantoea* strains (prepared as described above). Adult worms were washed off plates, washed twice with M9, transferred to slow killing plates seeded with PA14-GFP (Shapira and Tan, 2008), and further replenished with the respective *Pantoea* strains (50 ml 20-fold concentrated two-day culture). Following additional 40 to 48 hours at 25°C, 20-30 worms were picked off plates, washed twice with 1 mL M9 in an Eppendorf tube, paralyzed with 10mM levamisole and mounted on 2% to 4% agarose pads to be imaged using a Leica MZ16F stereoscope equipped with a QImaging MicroPublisher 5.0 camera. Images of worms of different experimental groups were all taken using identical exposure settings.

### Image Analysis

Quantification of fluorescence signal was achieved using a protocol implemented on the Fiji plugin within the ImageJ package v2.10/1.53c (Schindelin et al., 2012). Fluorescent signal per worm was calculated using Integrated Density values, following subtraction of background mean gray values and autofluorescence, and subsequently normalized for worm size.

To bring together results from different experiments, normalized fluorescent signal values per worm were additionally scaled by the median value in worms raised on OP50 of the respective experiment.

Normalized PA14-GFP signal values correlated well with the number of colonizing bacteria as evaluated by colony-forming unit (CFU) counts (**Supplementary Figure 1**).

### Paraformaldehyde-Killed Bacteria

Bacteria were killed by a one-hour incubation at 28°C with paraformaldehyde (PFA) at a final concentration of 0.5% as previously described (Beydoun et al., 2021). Dead bacteria were then washed five times, concentrated, OD adjusted and plated on the appropriate plates.

### CFU Counts

Adult worms raised at 20°C (72 hours) on PFM plates with lawns of the examined bacterial strains were rinsed off and washed as previously described with several extra washes (Dirksen et al., 2020). Three 10-worm samples were taken from each population and washed five times more with 1 mL M9/0.01% Triton. Each 10-worm sample was transferred in a 100 mL volume to tubes containing 10-15 zirconia beads and broken to release bacteria using a PowerLyser (30 sec, 4000 rpm). Lysates were serially diluted, plated on VRBG plates, and colonies appearing after 24 hours of incubation at 28°C were counted. Post-wash worm-free supernatant was plated in parallel to ascertain the lack of contaminating external bacteria.

### Bacterial Choice Assays

Worms were raised to young adulthood on OP50 lawns on PFM plates and shifted following three M9 washes to the center of test plates equidistantly spotted (5 uL volume, 1.5 cm from center) with tested strains at similar densities. In pairwise comparisons, plates contained two spots of each strain; worms at each spot were counted following 3 hours at 20°C, calculating a choice index (CI) as # Worms on strain A - #Worms on strain B/Total # of scored worms (Abada et al., 2009). In multiple strain plates, worms were offered a choice of each of the *Pantoea* strains as well as of OP50, and the percentage in each spot was evaluated after 3 hours.

### Statistical Analyses

Statistical evaluation was performed in R (v 3.6.3). Plots were generated using the ggplot2 R package (Villanueva and Chen, 2019).

## Results

### Worm Gut Commensals and Their Environmental Counterparts Frequently Promote Faster Development Than the Standard *E. coli* Food

**Figure 1:**
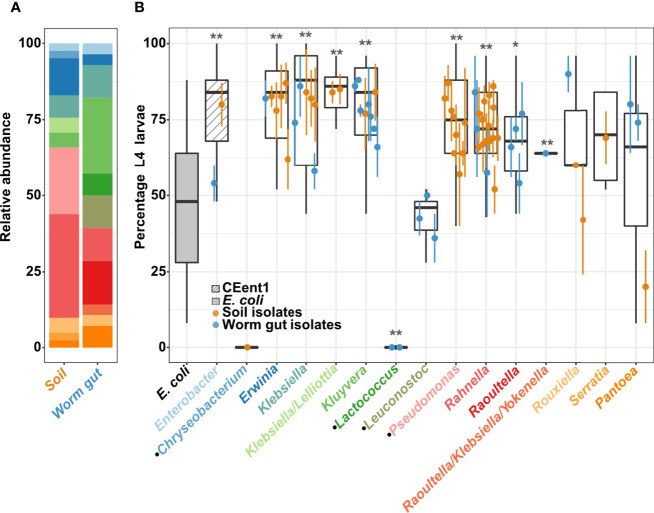
Effects of environmental and worm gut isolates on *C. elegans* development. **(A)** Bacterial isolates distribution. Relative abundance of genera recovered from soil microcosms and from worm guts, identified based on full length 16S Sanger sequencing and colored as in panel B *x*-axis (see also **Supplementary Table 1**). **(B)** Percentage of L4 larvae in worm populations raised from eggs for 40 hours at 25°C on environmental or gut bacterial isolates, as designated, grouped at the genus level; N=25/strain/experiment, dots represent averages (± SE) of 2 independent experiments. *E. coli* strain OP50 and *Enterobacter hormaechei* strain CEent1, in the gray and the hatched box, respectively, represent values from 41 experiments and provide reference for comparisons. Dotted lines represent the mean value for development on OP50, mean + SD and mean + 2 SD’s. Boxes extend from first to third quartiles, with black lines representing the median. *p < 0.05; **p < 0.001, in comparison to *E. coli* (Student’s t-test, corrected for multiple testing using Bonferroni). Black dots at *x*-axis labels indicate *non-Enterobacteriales* isolates.

To explore *C. elegans* preferences of bacteria from its environment, and bacterial contributions to host fitness, we isolated bacteria from worms raised in compost microcosms, as well as from their immediate environments. Microcosms were made of local soil composted with produce to emulate environments from which *C. elegans* has been previously isolated (Berg et al., 2016a). Bacteria were isolated by culturing on VRBG selective medium (see Methods), to focus on bacteria of the *Enterobacteriaceae* family/*Enterobacterales* order, which were previously shown to be a significant component of the worm gut microbiota and have diverse effects on host fitness (Berg et al., 2016a; 2016b; Samuel et al., 2016; Zhang et al., 2017; Slowinski et al., 2020). Culturing on rich media plates further supplemented isolation efforts with non-*Enterobacterales* isolates. Sixty-nine of the isolates were characterized by full-length 16S (identified at genus-level resolution, although not always unambiguously) and were employed in subsequent analysis (**Figure 1A**; **Supplementary Table S1**, Accession numbers OK487509-OK487574). Among the different genera, some were represented mostly (or exclusively) by environmental isolates (*Erwinia, Pseudomonas, Rahnella*), while others were represented primarily by gut isolates (*Kluyvera* and *Raoultella*). This may suggest preferred niches for the respective genera. However, it should be noted that bacteria of the *Pseudomonas* genus (which also grew on VRBG with a different colony morphology than *Enterobacterales*), here not found in worm guts, were previously isolated from worms (Montalvo-Katz et al., 2013; Schulenburg and Felix, 2017; Zimmermann et al., 2020).

Initial analysis examined the effects of individual bacterial isolates on host development, quantifying the percentage of worms reaching the last larval stage (L4) after 40 hours. Overall, most isolates (either gut or soil microcosm strains) of the examined genera promoted significantly faster development than the standard food *E. coli* strain OP50, and similar to the previously described *Enterobacter hormaechaei* CEent1 gut commensal (Berg et al., 2016b; Dirksen et al., 2020). In contrast, raising worms on most non-*Enterobacterales* genera, i.e., *Chryseobacterium, Leuconostoc* and *Lactococcus*, demonstrated slower or similarly-paced development, compared to OP50, in agreement with previous reports (Dirksen et al., 2016; Samuel et al., 2016; Dirksen et al., 2020).

Interestingly, among members of the *Erwiniaceae* genus *Pantoea* (a recent splinter off *Enterobacteriaceae*), gut isolates promoted significantly faster development than an environmental isolate (**Figure 1B** and **Supplementary Figure 2**). Isolates of the *Rouxiella* genus showed a similar distinction between gut and soil isolates, but not as significant as with *Pantoea* isolates. *Pantoea* bacteria, together with other *Enterobacterales* genera, are known to be part of the *C. elegans* microbiota (Berg et al., 2016a), and a previously identified *Pantoea* gut isolate, BIGb0393, which is part of the CeMBio community, has been shown to promote fast development in *C. elegans* despite not being a prolific colonizer (Dirksen et al., 2020). Our screen further suggests that a subset of *Pantoea* bacteria contributes to fast development in *C. elegans.*

### Gut *Pantoea* Offer Protection From Pathogenic Colonization by *Pseudomonas aeruginosa*

**Figure 2:**
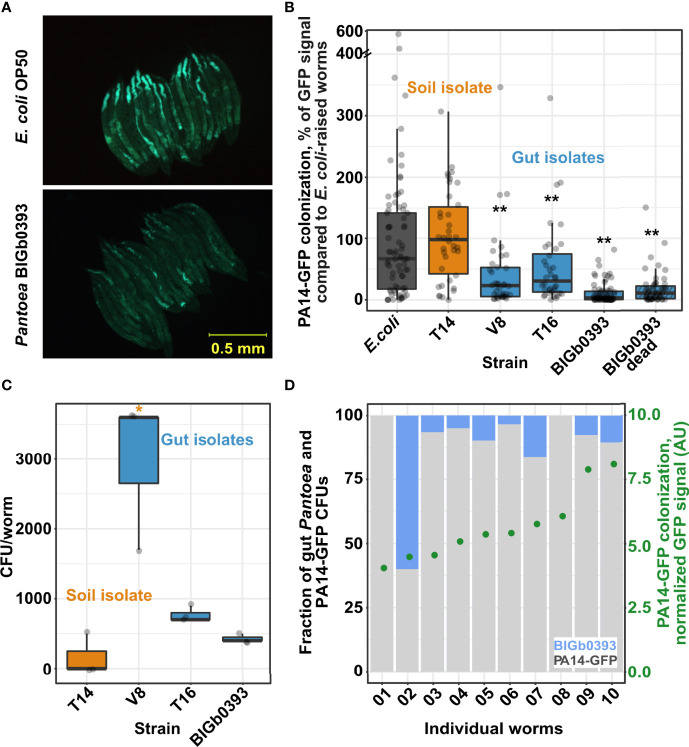
Gut isolates but not an environmental *Pantoea* strain provide protection from pathogenic colonization by *Pseudomonas aeruginosa* PA14. **(A)** Worm colonization by PA14-GFP following 43-45 hours of exposure. **(B)** Quantification of PA14-GFP fluorescent signal in images as in A; N=36-75 worms from two or three independent experiments. **(C)** Worm colonization by individual *Pantoea* strains. CFU counts were estimated in worms raised for 72 hours starting at the L1 stage. Shown are measurements performed in triplicate (N=10 worms each). B and **(C)** * and **, indicate *p* < 0.05 and *p* < 0.001, respectively (Student’s t-test). **(D)** Gut colonization in individual worms raised on BIGb0393 and shifted for 44 hours to *P. aeruginosa* PA14-GFP. No correlation is observed between *Pantoea* colonization (left *y*-axis, based on CFU counts), and PA14-GFP colonization [right *y*-axis, fluorescent signal, arbitrary units (AU)].

To expand examination of additional fitness measures that may be affected by *Pantoea* isolates, we tested their ability to prevent pathogenic colonization by the virulent *Pseudomonas aeruginosa* PA14. We found that worms raised on the three *Pantoea* strains recovered from the *C. elegans* gut (the previously described BIGb0393 and the two identified in this study (V8 and T16)) were more resistant to subsequent colonization by PA14-GFP than worms raised on *E. coli*, and additionally, more resistant than worms raised on a *Pantoea* strain isolated from the worm microcosm environment (T14) (**Figures 2A, B**). Subsequent analyses showed that gut *Pantoea* were better at colonizing the worm gut than the environmental strain (**Figure 2C**), similar to previous reports for BIGb0393 (Dirksen et al., 2020). However, better colonization, and presumably competition, did not seem to be at the basis of *Pantoea’s* ability to prevent infection, since CFU analysis of worms following PA14-GFP colonization (after 44 hours of exposure) identified only a few remaining *Pantoea* in the gut, which was not correlated with the extent of PA14-GFP colonization (**Figure 2D**), and since dead *Pantoea* were as capable of preventing PA14 colonization as live ones (**Figure 2B**). Together, these results suggest that *Pantoea* strains help prevent PA14 colonization but not through competition.

### Worms Prefer Beneficial Gut *Pantoea*

**Figure 3:**
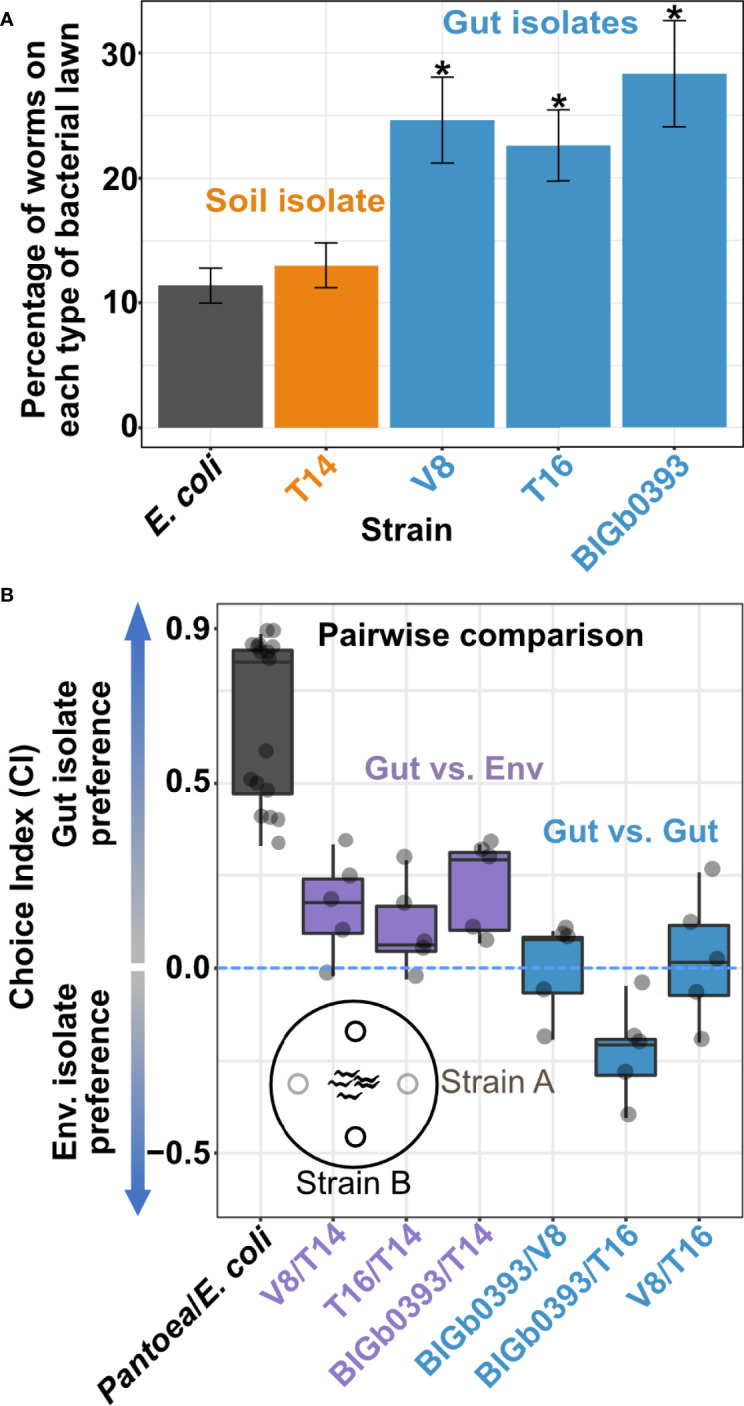
*C. elegans* shows preference towards beneficial *Pantoea.* **(A)** Multi-strain plates. Shown are percentages of young adult worms localized to spots of the designated strains after a three-hour incubation at 25°C. Shown are averages ± SEM for 10 plates (N=36-164 worms each). *p < 0.01 (Student’s t-test). **(B)** Pairwise preference comparisons. Shown are choice index values of young adult worms exposed to designated strains for 3 hours. Each dot represents an independent pairwise comparison between the designated *Pantoea* strains (N=21-123 worms per comparison), with boxes defining the 25 to 75 percentile values and lines representing the median value.

Our results suggested that the characterized beneficial *Pantoea* were better able to colonize the worm intestine, which is likely what made them gut commensals. However, behavioral mechanisms were also shown to affect worm interactions with bacteria. Worms are known to avoid pathogenic bacteria (Pradel et al., 2007) or show attraction to food bacteria that better support growth (Shtonda and Avery, 2006). We therefore investigated whether worms preferred specific *Pantoea* strains. Both multiple-choice and pairwise choice assays revealed significant preference of the beneficial *Pantoea* gut isolates over the environmental isolate or over control OP50 (**Figures 3A, B**). These results indicate that behavioral mechanisms further promote preference of beneficial commensals over a similar yet non-beneficial congeneric.

## Discussion

In exploring the adaptive capacities of environmentally-acquired commensals for *C. elegans* fitness, we identified members of the genus *Pantoea* as multi-purpose beneficial commensals. Gut *Pantoea* promoted fast development and further hindered pathogenic colonization. While the number of strains available for examination was rather low, it appears that benefits were limited to *Pantoea* isolated from the worm gut and were missing from a congeneric soil isolate. Gut *Pantoea* were better at colonizing the worm, and in addition, worms seemed to prefer them over the less beneficial soil *Pantoea* or the *E. coli* food bacteria. This behavior adds to previous reports of worms preferring bacteria that support their growth and development (Shtonda and Avery, 2006), by expanding such preference to bacteria that are beneficial beyond nutrition.

*C. elegans* behavioral food preferences are linked to its neuronal responses to external bacterial cues (e.g., bacterial odors), helping to pre-emptively avoid pathogenic bacteria and promoting interactions with beneficial ones. In addition, *C. elegans* can exhibit learned behaviors within one to three hours after sampling different food sources, where in some cases, consumption of preferred bacteria has been correlated with increased lifespan and, to a lesser extent, with increased growth rates (Troemel et al., 1997; Law et al., 2004; Shtonda and Avery, 2006; Sowa et al., 2015; Worthy et al., 2018). The capability of *C. elegans* to prefer beneficial microbes may be comparable to that observed in other eukaryotic models. In *Drosophila melanogaster*, for example, olfactory signals synthesized by gut-associated microbes were shown to modify host foraging behavior, egg laying, and nutritional as well as microbial preferences (Fischer et al., 2017; Wong et al., 2017). Unlike the microbial signals in the drosophila model, the molecular basis for *Pantoea* preference by *C. elegans* is yet unknown. However, an intriguing parallel in the effects that bacteria exerts on their host is presented by a recently described *Providencia* gut commensal, which was shown to promote worm growth and was able to attract worms by modulating host neurotransmission (Samuel et al., 2016; O’Donnell et al., 2020).

Colonization resistance conferred by gut bacteria can be due to direct antagonism of invaders (*i.e*., resource competition or metabolic warfare) or through induction of host immune responses (Kamada et al., 2013; Montalvo-Katz et al., 2013; Iatsenko et al., 2014; Granato et al., 2019). While the basis of *Pantoea*’s ability to promote such resistance is not known, other *Pantoea* species have been reported to produce antimicrobial molecules, effective, among others, against *Pseudomonas aeruginosa* (Williams and Stavrinides, 2020). In the example we identified, hindering of pathogen colonization was likely not due to competition, as BIGb0393 conferred protection from PA14 colonization even when dead. Another alternative is immune priming, which was previously shown to provide protection from PA14 infection through activation of the p38 MAPK pathway (Montalvo-Katz et al., 2013). Other studies have shown that immune priming can be conferred by heat-killed bacteria (Lee et al., 2015; Yuen and Ausubel, 2018), suggesting that immune activation may be triggered by recognition of heat-labile microbe-associated molecular patterns (MAMPs), in line with previous studies (Twumasi-Boateng and Shapira, 2012). Lastly, it is also possible that the nutritional value of *Pantoea*, similar in live bacteria as in dead ones, makes for healthier worms, which can then better withstand infection. However, generally better health is more likely to contribute to long-term tolerance of infection and survival than on the initial stages of colonization, which are dependent on specific immune pathways.

The expansion in the reported abilities of *Pantoea* strains to accelerate development and contribute to host fitness present them as important members of the worm gut microbiota. The difference in the effects of, and host affinity to, some members of this taxa (gut isolates) compared to others (the soil isolate), distinguishes this genus from other examined genera. This, together with what appears to be mutual adaptations, including better ability of beneficial *Pantoea* to colonize the worm gut (compared to a non-beneficial congeneric) and host preference of its cognate commensals, suggest long-standing interactions between the worm host and its *Pantoea* gut commensals. How these interactions have evolved and how tightly they link the fitness of the two partners, remains to be further studied. Furthermore, research into *C. elegans* interactions with additional *Pantoea* strains is required to ascertain the paradigm proposed here and to provide further support.

## Data Availability Statement

The datasets presented in this study can be found in online repositories. The names of the repository/repositories and accession number(s) can be found in the article/**Supplementary Material.**

## Author Contributions

RC, OMPC, and MS contributed to the conception of the study and the preparation of the manuscript. MM conducted soil microcosm experiments, isolated bacteria and analyzed their effects on host development. OMPC and RC lead colonization experiments with pathogens. OMPC conducted preference assays. OMPC, RC, and BP contributed to designing colonization experiments, data acquisition, image quantification, and data analysis and interpretation. VN contributed to valuable experiments and results that were omitted in the final version. All authors contributed to the article and approved the submitted version.

## Funding

Work described in this manuscript was supported by NIH grants R01OD024780 and R01AG061302.

## Conflict of Interest

The authors declare that the research was conducted in the absence of any commercial or financial relationships that could be construed as a potential conflict of interest.

The reviewer MP declared a shared affiliation with the authors to the handling editor at the time of review.

## Publisher’s Note

All claims expressed in this article are solely those of the authors and do not necessarily represent those of their affiliated organizations, or those of the publisher, the editors and the reviewers. Any product that may be evaluated in this article, or claim that may be made by its manufacturer, is not guaranteed or endorsed by the publisher.
