# Supplementary material for: Host Preference of Beneficial Commensals in a Microbially-Diverse Environment
Source: Front Cell Infect Microbiol. 2022 Jun 15;12:795343. doi: 10.3389/fcimb.2022.795343 (PMC9240469; doi:10.3389/fcimb.2022.795343)
Supplement: Supplementary file 1 [file DataSheet_1.pdf]

Supplementary figures

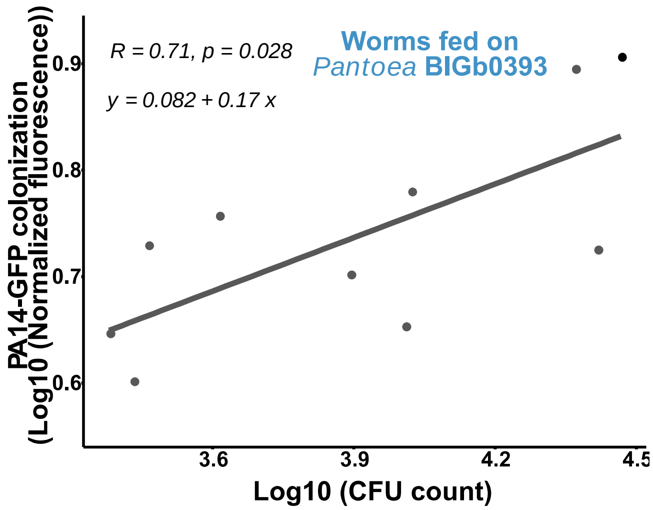

Figure S1. Significant correlation between *Pseudomonas* colonization levels and its CFU counts. Correlation between *Pseudomonas* PA14 colonization levels and its CFU counts in worms raised on the protective *Pantoea* strain BIGb0393. Fluorescence was estimated in individual worms (N=10) after 43 hours of exposure to PA14-GFP.

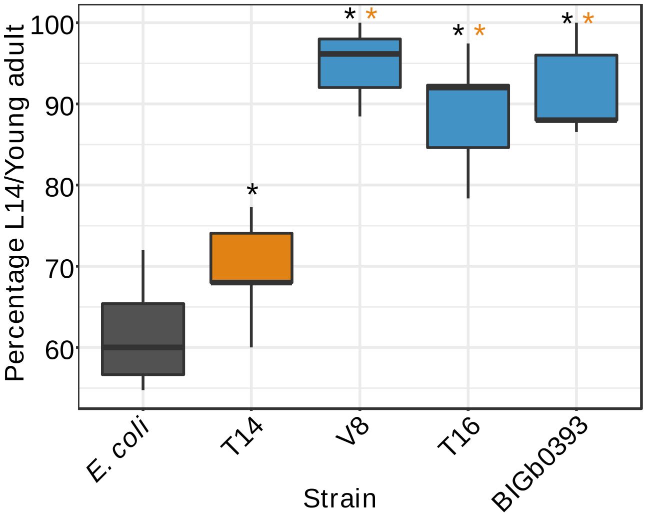

Figure S2. Development rate of *C. elegans* fed on *Pantoea* strains. The proportions of L4 larvae and young adults were measured after 28 hours incubation post L1 at 25ºC (25 to 57 worms per replica (N=3)). * indicate $p < 0.001$ (Student's t-test).
